# Aqua­chloridobis(diphenyl­glyoximato-κ^2^ *N*,*N*′)cobalt(III) dihydrate

**DOI:** 10.1107/S1600536811014280

**Published:** 2011-04-22

**Authors:** Parthasarathy Meera, Madhavan Amutha Selvi, Arunachalam Dayalan

**Affiliations:** a Department of Chemistry, Loyola College (Autonomous), Sterling Road, Nungambakkam, Chennai 600 034, Tamil Nadu, India

## Abstract

The asymmetric unit of the title complex, [Co(C_14_H_11_N_2_O_2_)_2_Cl(H_2_O)]·2H_2_O or [Co(dpgH)_2_Cl(H_2_O)]·2H_2_O, where dpgH^−^ is diphenyl glyoximate, consists of one-half of a [Co(dpgH)_2_Cl(H_2_O)] complex and one solvent water mol­ecule. The complex is completed through inversion symmetry, with the Co^III^ atom situated at the centre of symmetry. The coordination geometry around the Co^III^ atom is distorted octa­hedral with the four N atoms of the two dpgH^−^ ligands forming an approximate square plane with N—Co—N bite angles of 81.13 (14) and 98.87 (14)°. The Cl^−^ ligand and the water mol­ecule are disordered in a 1:1 ratio and are in the axial positions, almost perpendicular to the plane of the glyoximate ligands [O—Co—Cl = 175.3 (10)°]. The two glyoximate ligands are linked by strong intra­molecular O—H⋯O hydrogen bonds. In addition, O—H⋯O inter­actions involving the solvent water mol­ecules and O—H⋯N hydrogen-bonding inter­actions are also observed. The solvent water mol­ecule is disordered over five positions with different occupancies.

## Related literature

For related complexes, see: Gupta *et al.* (2003 ▶); Randaccio (1999 ▶); Brown & Satyanarayana (1992 ▶); Gilaberte *et al.* (1988 ▶). For the nature of equatorial ligands, see: Varhelyi *et al.* (1999 ▶). For similar structures, see: Meera *et al.* (2009 ▶). For details of the synthesis, see: Toscano *et al.* (1983 ▶); Gupta *et al.* (2001 ▶). For spectroscopic studies related to the complex, see: Gupta *et al.* (2004 ▶); Lopez *et al.* (1992 ▶); Silverstein & Bassler (1984 ▶); Mandal & Gupta (2005 ▶).
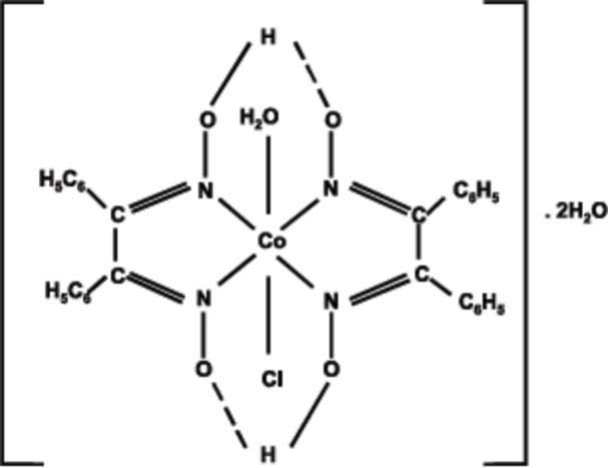


## Experimental

### 

#### Crystal data

[Co(C_14_H_11_N_2_O_2_)_2_Cl(H_2_O)]·2H_2_O
*M* *_r_* = 626.92
Monoclinic, 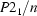

*a* = 12.0709 (4) Å
*b* = 5.9689 (2) Å
*c* = 21.9224 (5) Å
β = 104.770 (1)°
*V* = 1527.32 (8) Å^3^
*Z* = 2
Mo *K*α radiation
μ = 0.70 mm^−1^
*T* = 293 K
0.30 × 0.20 × 0.20 mm

#### Data collection

Bruker APEXII CCD diffractometer
Absorption correction: multi-scan (*SADABS*; Bruker, 1999 ▶) *T*_min_ = 0.761, *T*_max_ = 0.861
13610 measured reflections
2682 independent reflections
2431 reflections with *I* > 2σ(*I*)
*R*_int_ = 0.030

#### Refinement

*R*[*F* ^2^ > 2σ(*F* ^2^)] = 0.062
*wR*(*F* ^2^) = 0.199
*S* = 1.25
2682 reflections
215 parameters
1 restraint
H-atom parameters constrained
Δρ_max_ = 0.92 e Å^−3^
Δρ_min_ = −0.53 e Å^−3^

### 

Data collection: *APEX2* (Bruker, 2004 ▶); cell refinement: *SAINT* (Bruker, 2004 ▶); data reduction: *SAINT*; program(s) used to solve structure: *SIR92* (Altomare *et al.*, 1993 ▶); program(s) used to refine structure: *SHELXL97* (Sheldrick, 2008 ▶); molecular graphics: *ORTEP-3* (Farrugia, 1997 ▶) and *Mercury* (Macrae *et al.*, 2008 ▶); software used to prepare material for publication: *PLATON* (Spek, 2009 ▶).

## Supplementary Material

Crystal structure: contains datablocks global, I. DOI: 10.1107/S1600536811014280/wm2477sup1.cif

Structure factors: contains datablocks I. DOI: 10.1107/S1600536811014280/wm2477Isup2.hkl

Additional supplementary materials:  crystallographic information; 3D view; checkCIF report

## Figures and Tables

**Table 1:** Selected bond lengths (Å)

Co1—N2^i^	1.891 (3)
Co1—N1^i^	1.894 (3)
Co1—O3	1.95 (3)
Co1—Cl1	2.214 (11)

Symmetry code: (i) 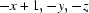.

**Table 2:** Hydrogen-bond geometry (Å, °)

*D*—H⋯*A*	*D*—H	H⋯*A*	*D*⋯*A*	*D*—H⋯*A*
O2—H2*A*⋯O1^i^	0.82	1.68	2.477 (4)	162
O2—H2*A*⋯N1^i^	0.82	2.40	2.999 (4)	130
O4*A*⋯O3^ii^			2.592 (4)	

Symmetry codes: (i) 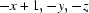; (ii) 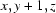.

**Fig. 1.**
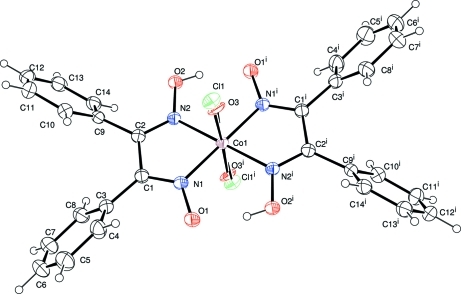
ORTEP representation of the complex drawn at the 30% probability level with the atom labelling scheme. [Symmetry Code: (i) 1-x, -y, -z].

**Fig. 2.**
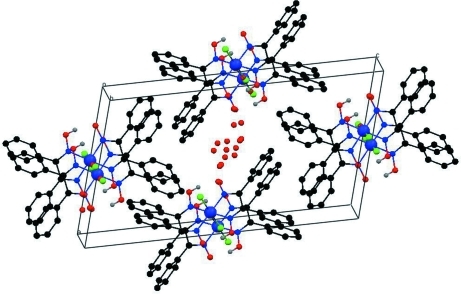
Packing of the complex in the unit cell with the disordered water occupying the intermolecular voids. The hydrogen atoms bound to aromatic carbons have been omitted for clarity.

## supplementary crystallographic information

### Comment

The dioxime complexes of cobalt(III), known as cobaloximes, and their derivatives have been found to mimic vitamin-B_12_ coenzyme. The studies on steric and electronic effects of cobaloximes helped in the successful design of novel derivatives with desired properties (Gupta *et al.*, 2003; Randaccio, 1999; Brown & Satyanarayana, 1992; Gilaberte *et al.*, 1988). Among the stereoisomeric benzildioximes (*syn*, *amphi*, *anti*), only the *anti* isomer shows chelation properties towards transition metal ions (Varhelyi *et al.*, 1999).

In the structure of the title compound, [Co(C_14_H_11_N_2_O_2_)_2_Cl(H_2_O)]^.^2H_2_O, or [Co(dpgH)_2_Cl(H_2_O)]^.^2H_2_O, where dpgH^-^ = diphenyl glyoximate, two halves of the complex molecule are related through inversion symmetry with Co^III^ situated at the centre of symmetry. The coordination geometry around Co^III^ is a slightly distorted octahedron (Fig. 1) with the four N atoms of the dpgH^-^ ligand forming an approximate square plane. The bite angles N1—Co—N2 of the equatorial ligands are 81.13 (14) and 98.87 (14)°, respectively. The Cl^-^ ligand and the water molecule are in axial positions and are disordered in a 1:1 ratio. They are almost perpendicular to the plane containing the equatorial dpgH^-^ ligand (O3—Co1—Cl1 = 175.3 (10)°). The two glyoximate ligands are linked by strong intramolecular O—H···O hydrogen bonds. In addition, O1—H1···N2 hydrogen bonding interaction is also observed (Fig. 2). A similar interaction was observed for a related complex (Meera *et al.*, 2009). The lattice water molecule (O4) is disordered over five positions with different occupancies. Although the H positions of the disordered water molecules could not be located, close O···O interactions suggest likewise an involvement in hydrogen bonding (Table 2).

### Experimental

Cobalt(II) chloride hexahydrate was thoroughly ground and mixed with diphenylglyoxime in a 1:2 molar ratio in an aqueous solution of acetone. The reaction mixture was stirred for five hours at an elevated temperature (Toscano *et al.*, 1983; Gupta *et al.*, 2001). The resulting brown mass was filtered, washed with acetone, ether and dried in a desiccator. Brown coloured crystals appeared in two to three days on slow evaporation of the saturated solution of the complex in ethanol. Elemental analysis, obtained by analytical method, agreed well with the theoretical data expected for the formula of the complex, C_28_H_28_N_4_O_7_ClCo. Anal., % (calc., %): C 53.97 (53.58); H 4.94 (4.47); N 9.05 (8.93). The C═N stretching vibration of oxime in its complex was observed at 1385 cm^-1^ and the intramolecular hydrogen bonded –OH around 3140 cm^-1^. A moderate peak around 1090 cm^-1^ may be assigned to the C=N—O stretching of the oxime. The band around 540 cm^-1^ could be attributed to cobalt(III)-nitrogen stretching. The ^1^H NMR spectrum of the complex in acetone-d_6_ shows three different signals corresponding to the three different aromatic protons of the diphenylglyoximate (Gupta *et al.*, 2004; Lopez *et al.*, 1992). The H atoms in the second and the sixth position of the benzene ring of the diphenylglyoximate show a doublet at 7.2 p.p.m., while the third and fifth H atoms show a triplet at 7.4 p.p.m.. Similarly, the fourth one gives a triplet at 7.3 p.p.m..The oxime –OH protons resonate at 9.1 p.p.m.. A singlet around 8.5 p.p.m. represents the protons of the –OH group of the aqua ligand (Silverstein & Bassler, 1984; Mandal & Gupta, 2005).

### Refinement

The O atom of the solvent water molecule in the lattice is disordered over five positions O4A, O4B, O4C, O4D, O4E with different site occupancy factor. The refinement of occupancy by means of free variable in each case is 0.302, 0.250, 0.131, 0.198 and 0.119 for O4A, O4B, O4C, O4D and O4E, respectively. The O atoms of water were refined anisotropically with equal anisotropic displacement parameters. The disordered chloride (Cl1) and oxygen (O3) atom sharing the axial position were refined with equal site occupancies of 1:1. The H atoms bound to aromatic carbon were constrained to ride on their parent atom with d(C—H) = 0.93Å and *U*_iso_(H) =1.2*U*_equ_(C). The position of the H atom bound to the hydroxyl group was identified from the difference in the electron density map and constrained to a distance of d(O2—H2) = 0.92 (1) Å. H positions of the positionally disordered lattice water molecules could not be found from difference maps and were eventually omitted from refinement.

### Crystal data

**Table:**

[Co(C_14_H_11_N_2_O_2_)_2_Cl(H_2_O)]·2H_2_O	*F*(000) = 648
*M**_r_* = 626.92	*D*_x_ = 1.363 Mg m^−^^3^
Monoclinic, *P*2_1_/*n*	Mo *K*α radiation, λ = 0.71073 Å
Hall symbol: -P 2yn	Cell parameters from 2441 reflections
*a* = 12.0709 (4) Å	θ = 2.8–25.0°
*b* = 5.9689 (2) Å	µ = 0.70 mm^−^^1^
*c* = 21.9224 (5) Å	*T* = 293 K
β = 104.770 (1)°	Block, brown
*V* = 1527.32 (8) Å^3^	0.30 × 0.20 × 0.20 mm
*Z* = 2	

### Data collection

**Table:**

Bruker APEXII CCD diffractometer	2682 independent reflections
Radiation source: fine-focus sealed tube	2431 reflections with *I* > 2σ(*I*)
graphite	*R*_int_ = 0.030
ω and φ scan	θ_max_ = 25.0°, θ_min_ = 1.8°
Absorption correction: multi-scan (*SADABS*; Bruker, 1999)	*h* = −14→14
*T*_min_ = 0.761, *T*_max_ = 0.861	*k* = −7→7
13610 measured reflections	*l* = −25→26

### Refinement

**Table:**

Refinement on *F*^2^	Secondary atom site location: difference Fourier map
Least-squares matrix: full	Hydrogen site location: inferred from neighbouring sites
*R*[*F*^2^ > 2σ(*F*^2^)] = 0.062	H-atom parameters constrained
*wR*(*F*^2^) = 0.199	*w* = 1/[σ^2^(*F*_o_^2^) + (0.1021*P*)^2^ + 2.2574*P*] where *P* = (*F*_o_^2^ + 2*F*_c_^2^)/3
*S* = 1.25	(Δ/σ)_max_ < 0.001
2682 reflections	Δρ_max_ = 0.92 e Å^−^^3^
215 parameters	Δρ_min_ = −0.53 e Å^−^^3^
1 restraint	Extinction correction: *SHELXL97* (Sheldrick, 2008), Fc^*^=kFc[1+0.001xFc^2^λ^3^/sin(2θ)]^-1/4^
Primary atom site location: structure-invariant direct methods	Extinction coefficient: 0.025 (3)

### Special details

**Table:**

Geometry. All e.s.d.'s (except the e.s.d. in the dihedral angle between two l.s. planes) are estimated using the full covariance matrix. The cell e.s.d.'s are taken into account individually in the estimation of e.s.d.'s in distances, angles and torsion angles; correlations between e.s.d.'s in cell parameters are only used when they are defined by crystal symmetry. An approximate (isotropic) treatment of cell e.s.d.'s is used for estimating e.s.d.'s involving l.s. planes.
Refinement. Refinement of *F*^2^ against ALL reflections. The weighted *R*-factor *wR* and goodness of fit *S* are based on *F*^2^, conventional *R*-factors *R* are based on *F*, with *F* set to zero for negative *F*^2^. The threshold expression of *F*^2^ > σ(*F*^2^) is used only for calculating *R*-factors(gt) *etc*. and is not relevant to the choice of reflections for refinement. *R*-factors based on *F*^2^ are statistically about twice as large as those based on *F*, and *R*- factors based on ALL data will be even larger.

### Fractional atomic coordinates and isotropic or equivalent isotropic displacement parameters (Å^2^)

**Table:**

	*x*	*y*	*z*	*U*_iso_*/*U*_eq_	Occ. (<1)
C1	0.3917 (4)	0.0079 (7)	0.0955 (2)	0.0349 (9)	
C2	0.5064 (3)	0.0989 (7)	0.12232 (19)	0.0342 (9)	
C3	0.3003 (4)	0.0027 (8)	0.1290 (2)	0.0404 (11)	
C4	0.2296 (4)	−0.1819 (9)	0.1254 (2)	0.0519 (12)	
H4	0.2423	−0.3092	0.1037	0.062*	
C5	0.1405 (5)	−0.1770 (12)	0.1539 (3)	0.0668 (16)	
H5	0.0927	−0.3009	0.1508	0.080*	
C6	0.1215 (5)	0.0054 (12)	0.1864 (3)	0.0677 (18)	
H6	0.0610	0.0062	0.2055	0.081*	
C7	0.1911 (5)	0.1880 (11)	0.1913 (3)	0.0619 (15)	
H7	0.1784	0.3131	0.2139	0.074*	
C8	0.2809 (4)	0.1867 (9)	0.1625 (2)	0.0482 (11)	
H8	0.3284	0.3112	0.1660	0.058*	
C9	0.5534 (3)	0.1512 (8)	0.19009 (18)	0.0353 (9)	
C10	0.5471 (4)	−0.0079 (8)	0.2349 (2)	0.0457 (11)	
H10	0.5078	−0.1412	0.2227	0.055*	
C11	0.5994 (5)	0.0313 (11)	0.2977 (2)	0.0576 (14)	
H11	0.5969	−0.0767	0.3279	0.069*	
C12	0.6550 (4)	0.2299 (11)	0.3153 (2)	0.0604 (16)	
H12	0.6900	0.2559	0.3576	0.073*	
C13	0.6600 (4)	0.3909 (10)	0.2718 (2)	0.0519 (13)	
H13	0.6972	0.5259	0.2844	0.062*	
C14	0.6092 (4)	0.3513 (8)	0.2090 (2)	0.0432 (11)	
H14	0.6125	0.4599	0.1791	0.052*	
N1	0.3780 (3)	−0.0578 (6)	0.03761 (16)	0.0335 (8)	
N2	0.5663 (3)	0.1165 (6)	0.08134 (15)	0.0325 (8)	
O1	0.2790 (2)	−0.1374 (6)	0.00398 (14)	0.0455 (8)	
O2	0.6748 (2)	0.1866 (6)	0.09899 (14)	0.0416 (8)	
H2A	0.7025	0.1849	0.0685	0.10 (3)*	
Co1	0.5000	0.0000	0.0000	0.0294 (3)	
Cl1	0.5813 (10)	−0.3227 (16)	0.0360 (5)	0.0429 (13)	0.50
O3	0.564 (2)	−0.296 (4)	0.0254 (12)	0.044 (7)	0.50
O4A	0.7645 (12)	0.624 (3)	0.0072 (7)	0.084 (3)	0.302 (9)
O4B	0.9669 (16)	0.417 (4)	0.0424 (9)	0.084 (3)	0.250 (10)
O4C	0.875 (3)	0.618 (7)	0.0176 (17)	0.084 (3)	0.131 (9)
O4D	1.023 (2)	0.437 (5)	−0.0007 (13)	0.084 (3)	0.198 (9)
O4E	1.002 (3)	0.295 (9)	0.028 (2)	0.084 (3)	0.119 (10)

### Atomic displacement parameters (Å^2^)

**Table:**

	*U*^11^	*U*^22^	*U*^33^	*U*^12^	*U*^13^	*U*^23^
C1	0.033 (2)	0.038 (2)	0.034 (2)	−0.0038 (17)	0.0086 (17)	−0.0021 (16)
C2	0.031 (2)	0.037 (2)	0.034 (2)	−0.0015 (17)	0.0082 (17)	0.0000 (17)
C3	0.033 (2)	0.054 (3)	0.033 (2)	−0.0056 (19)	0.0075 (18)	−0.0005 (18)
C4	0.054 (3)	0.059 (3)	0.046 (3)	−0.016 (2)	0.019 (2)	−0.004 (2)
C5	0.054 (3)	0.087 (4)	0.064 (3)	−0.030 (3)	0.023 (3)	0.003 (3)
C6	0.045 (3)	0.109 (5)	0.057 (3)	−0.008 (3)	0.027 (3)	−0.003 (3)
C7	0.049 (3)	0.083 (4)	0.058 (3)	0.010 (3)	0.022 (2)	−0.009 (3)
C8	0.039 (2)	0.058 (3)	0.050 (3)	−0.002 (2)	0.015 (2)	−0.005 (2)
C9	0.0275 (19)	0.046 (2)	0.033 (2)	0.0019 (18)	0.0090 (16)	−0.0058 (18)
C10	0.041 (3)	0.057 (3)	0.041 (2)	0.002 (2)	0.013 (2)	−0.001 (2)
C11	0.053 (3)	0.083 (4)	0.036 (3)	0.013 (3)	0.011 (2)	0.009 (2)
C12	0.038 (3)	0.104 (5)	0.035 (2)	0.008 (3)	0.002 (2)	−0.020 (3)
C13	0.038 (2)	0.067 (3)	0.050 (3)	−0.003 (2)	0.011 (2)	−0.023 (3)
C14	0.035 (2)	0.053 (3)	0.043 (2)	−0.005 (2)	0.0112 (18)	−0.008 (2)
N1	0.0282 (17)	0.0370 (18)	0.0344 (18)	−0.0050 (14)	0.0061 (14)	−0.0023 (15)
N2	0.0278 (17)	0.0378 (19)	0.0312 (16)	−0.0044 (14)	0.0061 (13)	−0.0015 (14)
O1	0.0311 (15)	0.065 (2)	0.0411 (16)	−0.0167 (15)	0.0101 (13)	−0.0096 (15)
O2	0.0272 (15)	0.060 (2)	0.0369 (15)	−0.0128 (14)	0.0064 (12)	−0.0088 (14)
Co1	0.0256 (5)	0.0331 (5)	0.0286 (5)	−0.0046 (3)	0.0054 (3)	−0.0011 (3)
Cl1	0.044 (3)	0.0374 (18)	0.041 (3)	0.0015 (18)	−0.001 (3)	0.004 (2)
O3	0.027 (8)	0.064 (12)	0.031 (8)	−0.003 (7)	−0.012 (5)	0.016 (5)
O4A	0.062 (6)	0.103 (9)	0.087 (7)	0.019 (6)	0.021 (5)	0.004 (6)
O4B	0.062 (6)	0.103 (9)	0.087 (7)	0.019 (6)	0.021 (5)	0.004 (6)
O4C	0.062 (6)	0.103 (9)	0.087 (7)	0.019 (6)	0.021 (5)	0.004 (6)
O4D	0.062 (6)	0.103 (9)	0.087 (7)	0.019 (6)	0.021 (5)	0.004 (6)
O4E	0.062 (6)	0.103 (9)	0.087 (7)	0.019 (6)	0.021 (5)	0.004 (6)

### Geometric parameters (Å, °)

**Table:**

C1—N1	1.298 (6)	C11—C12	1.368 (9)
C1—C2	1.463 (6)	C11—H11	0.9300
C1—C3	1.473 (6)	C12—C13	1.366 (8)
C2—N2	1.294 (5)	C12—H12	0.9300
C2—C9	1.482 (6)	C13—C14	1.377 (6)
C3—C8	1.375 (7)	C13—H13	0.9300
C3—C4	1.383 (7)	C14—H14	0.9300
C4—C5	1.375 (7)	N1—O1	1.323 (4)
C4—H4	0.9300	N1—Co1	1.894 (3)
C5—C6	1.353 (9)	N2—O2	1.334 (4)
C5—H5	0.9300	N2—Co1	1.891 (3)
C6—C7	1.363 (9)	O2—H2A	0.8200
C6—H6	0.9300	Co1—N2^i^	1.891 (3)
C7—C8	1.386 (7)	Co1—N1^i^	1.894 (3)
C7—H7	0.9300	Co1—O3	1.95 (3)
C8—H8	0.9300	Co1—O3^i^	1.95 (3)
C9—C14	1.382 (6)	Co1—Cl1	2.214 (11)
C9—C10	1.382 (6)	Co1—Cl1^i^	2.214 (11)
C10—C11	1.381 (7)	O4D—O4D^ii^	0.94 (4)
C10—H10	0.9300		
			
O4A···O3^iii^	2.592 (4)	O4A···CL1^iii^	2.470 (2)
O4A···O1^i^	2.951 (2)		
			
N1—C1—C2	112.1 (4)	C13—C14—C9	120.5 (5)
N1—C1—C3	123.8 (4)	C13—C14—H14	119.8
C2—C1—C3	124.0 (4)	C9—C14—H14	119.8
N2—C2—C1	113.0 (4)	C1—N1—O1	121.7 (3)
N2—C2—C9	122.6 (4)	C1—N1—Co1	116.8 (3)
C1—C2—C9	124.2 (4)	O1—N1—Co1	121.0 (3)
C8—C3—C4	118.7 (4)	C2—N2—O2	120.4 (3)
C8—C3—C1	120.1 (4)	C2—N2—Co1	116.6 (3)
C4—C3—C1	121.2 (4)	O2—N2—Co1	122.5 (2)
C5—C4—C3	120.0 (5)	N2—O2—H2A	109.5
C5—C4—H4	120.0	N2—Co1—N2^i^	179.998 (1)
C3—C4—H4	120.0	N2—Co1—N1	81.13 (14)
C6—C5—C4	120.9 (5)	N2^i^—Co1—N1	98.87 (14)
C6—C5—H5	119.5	N2—Co1—N1^i^	98.87 (14)
C4—C5—H5	119.5	N2^i^—Co1—N1^i^	81.13 (14)
C5—C6—C7	120.1 (5)	N1—Co1—N1^i^	180.00 (17)
C5—C6—H6	120.0	N2—Co1—O3	91.3 (7)
C7—C6—H6	120.0	N2^i^—Co1—O3	88.7 (7)
C6—C7—C8	119.8 (5)	N1—Co1—O3	90.4 (9)
C6—C7—H7	120.1	N1^i^—Co1—O3	89.6 (9)
C8—C7—H7	120.1	N2—Co1—O3^i^	88.7 (7)
C3—C8—C7	120.5 (5)	N2^i^—Co1—O3^i^	91.3 (7)
C3—C8—H8	119.7	N1—Co1—O3^i^	89.6 (9)
C7—C8—H8	119.7	N1^i^—Co1—O3^i^	90.4 (9)
C14—C9—C10	119.5 (4)	O3—Co1—O3^i^	179.999 (2)
C14—C9—C2	121.0 (4)	N2—Co1—Cl1	86.6 (3)
C10—C9—C2	119.4 (4)	N2^i^—Co1—Cl1	93.4 (3)
C11—C10—C9	119.9 (5)	N1—Co1—Cl1	90.6 (3)
C11—C10—H10	120.1	N1^i^—Co1—Cl1	89.4 (3)
C9—C10—H10	120.1	O3—Co1—Cl1	4.7 (10)
C12—C11—C10	119.6 (5)	O3^i^—Co1—Cl1	175.3 (10)
C12—C11—H11	120.2	N2—Co1—Cl1^i^	93.4 (3)
C10—C11—H11	120.2	N2^i^—Co1—Cl1^i^	86.6 (3)
C13—C12—C11	121.3 (4)	N1—Co1—Cl1^i^	89.4 (3)
C13—C12—H12	119.4	N1^i^—Co1—Cl1^i^	90.6 (3)
C11—C12—H12	119.4	O3—Co1—Cl1^i^	175.3 (10)
C12—C13—C14	119.3 (5)	O3^i^—Co1—Cl1^i^	4.7 (10)
C12—C13—H13	120.3	Cl1—Co1—Cl1^i^	179.999 (1)
C14—C13—H13	120.3		
			
N1—C1—C2—N2	6.6 (5)	C1—C2—N2—O2	−176.8 (3)
C3—C1—C2—N2	−170.3 (4)	C9—C2—N2—O2	−0.8 (6)
N1—C1—C2—C9	−169.3 (4)	C1—C2—N2—Co1	−5.3 (5)
C3—C1—C2—C9	13.8 (7)	C9—C2—N2—Co1	170.7 (3)
N1—C1—C3—C8	−132.9 (5)	C2—N2—Co1—N2^i^	164 (6)
C2—C1—C3—C8	43.6 (6)	O2—N2—Co1—N2^i^	−25 (6)
N1—C1—C3—C4	44.5 (7)	C2—N2—Co1—N1	2.1 (3)
C2—C1—C3—C4	−139.0 (5)	O2—N2—Co1—N1	173.5 (3)
C8—C3—C4—C5	1.4 (7)	C2—N2—Co1—N1^i^	−177.9 (3)
C1—C3—C4—C5	−176.0 (5)	O2—N2—Co1—N1^i^	−6.5 (3)
C3—C4—C5—C6	−0.9 (8)	C2—N2—Co1—O3	−88.1 (10)
C4—C5—C6—C7	0.0 (9)	O2—N2—Co1—O3	83.2 (10)
C5—C6—C7—C8	0.4 (9)	C2—N2—Co1—O3^i^	91.9 (10)
C4—C3—C8—C7	−1.0 (7)	O2—N2—Co1—O3^i^	−96.8 (10)
C1—C3—C8—C7	176.4 (4)	C2—N2—Co1—Cl1	−89.0 (4)
C6—C7—C8—C3	0.1 (8)	O2—N2—Co1—Cl1	82.4 (4)
N2—C2—C9—C14	50.7 (6)	C2—N2—Co1—Cl1^i^	91.0 (4)
C1—C2—C9—C14	−133.8 (4)	O2—N2—Co1—Cl1^i^	−97.6 (4)
N2—C2—C9—C10	−125.5 (5)	C1—N1—Co1—N2	2.0 (3)
C1—C2—C9—C10	50.0 (6)	O1—N1—Co1—N2	174.3 (3)
C14—C9—C10—C11	−1.9 (7)	C1—N1—Co1—N2^i^	−178.0 (3)
C2—C9—C10—C11	174.4 (4)	O1—N1—Co1—N2^i^	−5.7 (3)
C9—C10—C11—C12	1.4 (7)	C1—N1—Co1—N1^i^	−133 (100)
C10—C11—C12—C13	−0.1 (8)	O1—N1—Co1—N1^i^	39 (100)
C11—C12—C13—C14	−0.8 (7)	C1—N1—Co1—O3	93.2 (8)
C12—C13—C14—C9	0.2 (7)	O1—N1—Co1—O3	−94.5 (8)
C10—C9—C14—C13	1.1 (6)	C1—N1—Co1—O3^i^	−86.8 (8)
C2—C9—C14—C13	−175.1 (4)	O1—N1—Co1—O3^i^	85.5 (8)
C2—C1—N1—O1	−177.4 (4)	C1—N1—Co1—Cl1	88.4 (4)
C3—C1—N1—O1	−0.5 (6)	O1—N1—Co1—Cl1	−99.2 (4)
C2—C1—N1—Co1	−5.1 (5)	C1—N1—Co1—Cl1^i^	−91.6 (4)
C3—C1—N1—Co1	171.8 (3)	O1—N1—Co1—Cl1^i^	80.8 (4)

Symmetry codes: (i) −*x*+1, −*y*, −*z*; (ii) −*x*+2, −*y*+1, −*z*; (iii) *x*, *y*+1, *z*.

### Hydrogen-bond geometry (Å, °)

**Table:**

*D*—H···*A*	*D*—H	H···*A*	*D*···*A*	*D*—H···*A*
O2—H2A···O1^i^	0.82	1.68	2.477 (4)	162.
O2—H2A···N1^i^	0.82	2.40	2.999 (4)	130.
O4A—···.O3^iii^	.	.	2.592 (4)	.

Symmetry codes: (i) −*x*+1, −*y*, −*z*; (iii) *x*, *y*+1, *z*.
